# Skeletal Muscle Metastasis in Papillary Thyroid Microcarcinoma Evaluated by F18-FDG PET/CT

**DOI:** 10.3390/diagnostics10020100

**Published:** 2020-02-12

**Authors:** Liviu Hitu, Calin Cainap, Dragos Apostu, Katalin Gabora, Eduard-Alexandru Bonci, Marius Badan, Alexandru Mester, Andra Piciu

**Affiliations:** 1 Iuliu Hatieganu University of Medicine and Pharmacy, 400012 Cluj-Napoca, Romania; Liviu.Hitu@umfcluj.ro (L.H.); gabora.katalin@yahoo.com (K.G.); bonci.eduard@gmail.com (E.-A.B.); badan_marius@yahoo.com (M.B.); 2 Department of Medical Oncology, Iuliu Hatieganu University of Medicine and Pharmacy, 400012 Cluj-Napoca, Romania; calincainap2015@gmail.com (C.C.); piciuandra@gmail.com (A.P.); 3 Department of Orthopedics and Traumatology, Iuliu Hatieganu University of Medicine and Pharmacy, 400012 Cluj-Napoca, Romania; apostudragos@yahoo.com; 4 Department of Oral Health, Iuliu Hatieganu University of Medicine and Pharmacy, 400012 Cluj-Napoca, Romania

**Keywords:** papillary thyroid carcinoma, papillary microcarcinoma, muscle metastasis, solitary muscle metastasis, FDG PET/CT, 18F-fludeoxyglucose positron emission tomography computed tomography, thyroglobulin, TENIS syndrome

## Abstract

Papillary thyroid cancer (PTC) is the most common type of thyroid malignancy and is characterized by slow growth and an indolent biological behavior. Papillary thyroid microcarcinoma is the PTC with the maximum size of the tumor <1cm, considered the most indolent form of thyroid cancer. PTC is usually metastasizes in cervical lymph nodes, lungs and bones and, less commonly, in brain or liver. Skeletal muscle metastases from PTC are extremely rare, a retrospective review of the literature revealed only 13 case reports. Among them, six cases are solitary skeletal muscle metastases, and seven are multiple metastases, most of them being associated with lung lesions. It seems that PTC is prone to metastasizing to the erector spinae and thigh muscles groups with unique cases located in trapezoid, biceps, deltoid, gastrocnemius and rectus abdominis muscles. Although extremely rare, one must bear in mind the fact that muscle metastasis from PTC is possible, and that is the reason we would like to discuss the existing clinical cases and to add a unique case of solitary skeletal muscle metastasis from papillary microcarcinoma.

## 1. Introduction

Papillary thyroid cancer (PTC) is the most common thyroid malignancy [1], and it is defined as a malignant epithelial tumor with evidence of follicular differentiation and a series of specific nuclear features [2]. Papillary thyroid microcarcinoma is the PTC with the maximum size of the tumor <1cm, considered the most indolent form of thyroid cancer. The incidence of PTC is increasing due to improved diagnostic methods such as ultrasound (US) with targeted fine-needle aspiration biopsy (FNAB) [3]. Cervical lymph nodes, lungs and bones are the most common metastatic [4] sites, brain, liver and skin involvement is less common. Distant metastases are usually diagnosed because of clinical symptoms or suspicious imaging/laboratory findings (abnormal uptake on post ablation WBS, or a positive finding on an FDG-PET/CT scan or cross-sectional study prompted by elevated thyroglobulin levels in patients whose post-ablation WBS is negative [5]. Literature data indicate that skeletal muscle metastases from PTC are extremely rare, with less than thirteen cases reported. Usually papillary microcarcinoma has an excellent prognosis [6]. To our knowledge, there has been only one report in the specialty literature of muscular metastasis from papillary microcarcinoma [7]. A unique case of metastatic thyroid papillary microcarcinoma to the gluteal muscle is presented, including a review of all thirteen cases reported in the literature.

## 2. The Available Evidence of the Existing Cases

Papillary thyroid cancer is considered to be a relatively indolent tumor; it has a slow progression with 10-year survival rates exceeding 90%–95% [8], but this may depend upon patient age. Only 5–10% of all cases will develop metastatic disease [2]. It’s spread is most lymphatic to cervical lymph nodes, and therefore distant metastases are a limited occurrence. Lungs and bone are the usual sites for distant metastases, rarely followed by liver and brain. Some other exceptional metastatic sites as sphenoid sinus, orbit, adrenal, kidney and ovary were reported [9].

**Figure 1:**
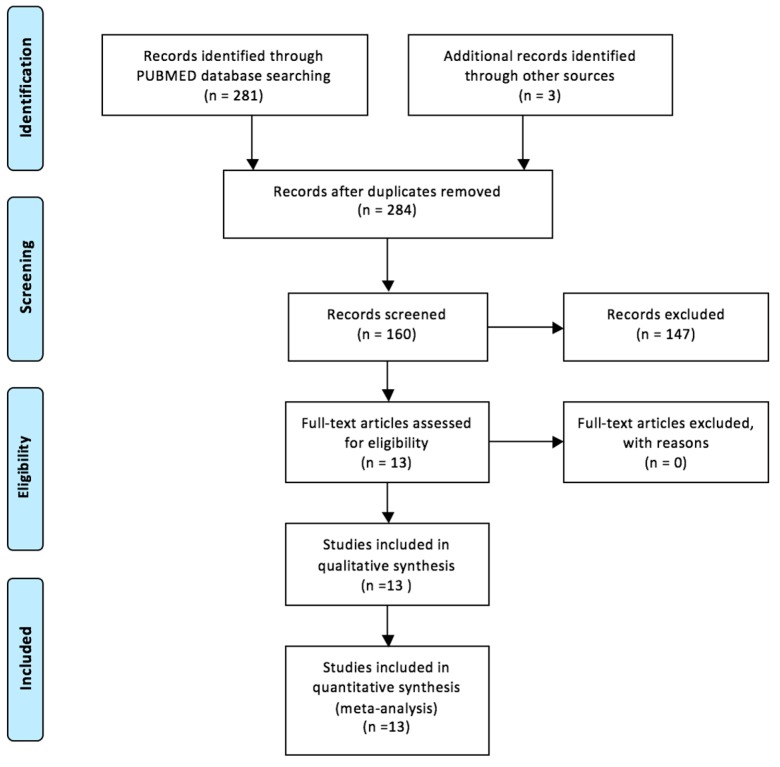
PRISMA flow diagram of selected studies.

We interrogated the PubMed database. To perform a reproducible search, we used several terms, keywords: “muscle metastasis thyroid”. We obtained 281 articles (clinical trial and review). We adjusted the search filters adding “full text”, “humans” and “English” so we have a selection of 157 articles. We applied the following inclusion criteria: the distant skeletal muscle metastasis (solitary or multiple) from papillary thyroid carcinoma. Also, we analyzed the bibliography of each article so we added 3 more studies, two of them were found on PubMed database and another through ResearchGate. Finally, we have a selection of 13 articles published between 2006–2016. The PRISMA flow diagram is presented in Figure 1.

Our retrospective review of the literature, show only thirteen cases of muscle metastasis arising from PTC Table 1. Out of 13 cases, only 4 patients are women, the median age of all patients being 66 years.

There is a special comment related to the high number of cases in male, comparing with female. In our review the ratio of male/female is 9/4, very different from the gender prevalence of differentiated thyroid cancer; worldwide it is known that thyroid cancer is more frequent in women, than in male. A possible argument, but not a clear explanation is that the aggressiveness of thyroid cancer is gender related, being more aggressive in males.

A possible explanation of rare muscle metastasis would be the hostile environment of lactic acid and continuous muscle motion [21]. There are only five cases described as a solitary muscle metastasis from PTC. Bae et al. reports a case of vastus medialis metastasis seen on a FDG-PET/CT scan as a focal uptake [10]. Panoussopoulos et al. describes a study of PTC with the metastatic site in the trapezoid muscle [17]. A case of PTC presenting as a solitary metastasis in the right arm muscle in an elderly hyperthyroid male patient was mentioned by Pucci et al. [18]. Similar to our study, the only case of papillary microcarcinoma skeletal metastasis found in the literature, is presented by Sarma et al., considering a 66 years old male left deltoid metastasis found on an FDG-PET/CT scan, after negative 131-iodine WBS while thyroglobulin level was 123.2 ng/mL [7]. Zhao et al. reported the case of rectus abdominis muscle metastasis from PTC identified by I-131 SPECT/CT as an incidental focus of abnormally increased I-131 uptake [20]. A very curious, particular case of two solitary muscle metastasis is presented by Caobelli et al., as a recurrence after seven years of disease free with 524 ng/mL serum TG, diagnosed on a FDG-PET/CT scan as two muscular distant lesions, right adductor longus and right iliopsoas [12]. The other seven cases reported as skeletal muscle metastasis associated with other sites are usually combined with lung metastasis [9,11,13,15]. The most common skeletal metastasis site appears to be the erector spinae [14,15,16,19] and thigh muscle group [10,11,12,13]. Unique cases of muscle metastasis sites were reported localized in trapezoid [17], arm biceps [18], deltoid [7], gastrocnemius [9], and rectus abdominis [20] muscles. In summary, to the best of our knowledge, this is the second reported case of distant solitary skeletal metastasis from papillary thyroid microcarcinoma. It’s very important to acknowledge that patients with growing Tg levels and negative I-131 WBS should be further investigated for possible dedifferentiation of the thyroid neoplasm. In case of thyroid carcinoma, despite the limited indication of 18F FDG-PET/CT, there is a very-well-defined place of PET/CT: biochemical evolution of the disease, with no clinical signs, increasing serological tumor markers, negative WBS I-131 or in other words- TENIS (Thyroglobulin Elevation Negative Iodine Scintigraphy) syndrome [22,23].

**Figure 2:**
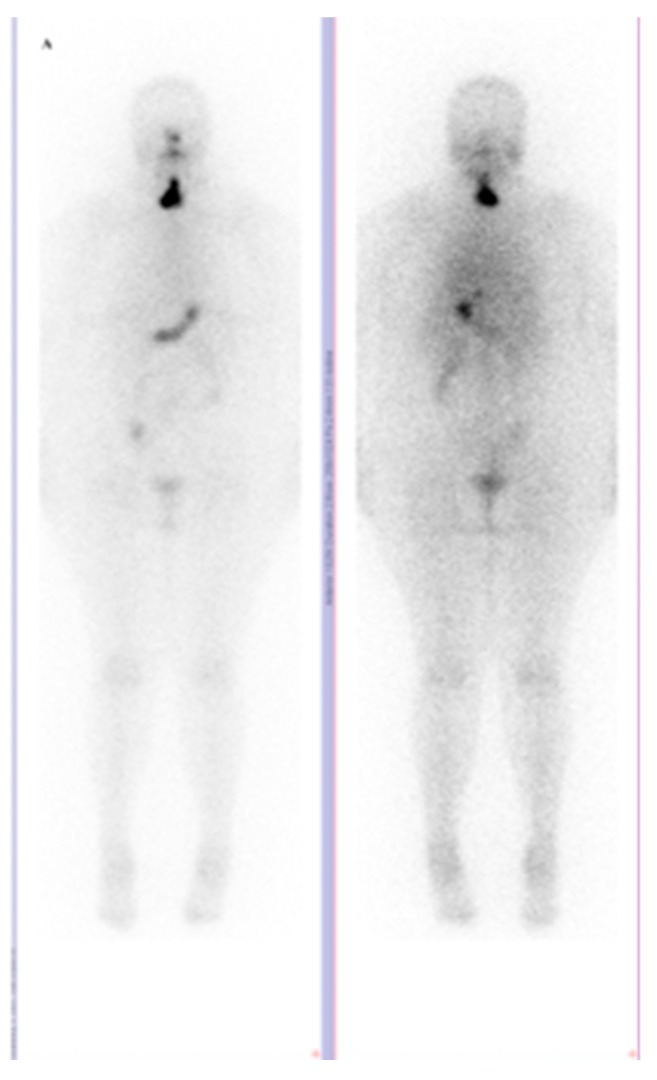
Whole Body Scan with I-131 sodium iodide (I-131 WBS) with 42,36 mCi (1,56 GBq) at 48 h postadministration, with thyroid remnant uptake (anterior **left**, posterior **right**) (2010).

Based the available evidence, we would like to add a case of a 58-year-old Caucasian woman, with the diagnosis of an incidental multiple papillary thyroid microcarcinoma, without any histological pattern of aggressiveness, operated by total thyroidectomy at the end of 2009. Postsurgery serum thyroglobulin (Tg) level was undetectable (<0.1 ng/mL) in the condition of correct TSH level stimulation (68.18 µUI/mL), but her thyroglobulin anti-bodies (TgAB) were still positive (331 UI/mL).The patient underwent radioiodine therapy (1.56 GBq) according to the guidelines of the moment [5] in February 2010, with the post-therapy whole body scan (WBS) showing thyroid remnant (Figure 2) and also having thyroid hormone replacement with correct TSH suppression. Eight months after therapy, the neck ultrasound was negative, Tg level undetectable, the TgAB levels normalized (108 UI/mL, normal <115), and the patient was in complete remission and disease free for eight years. In 2018, on a routine check-up, Tg level was 9.49 ng/mL and neck US revealed a solitary left latero-cervical lymphadenopathy of 18/10.5/9.5 mm with high vascularization for which she underwent unilateral neck dissection. Histological examination found no metastases, the result being histiocytosis.

**Figure 3:**
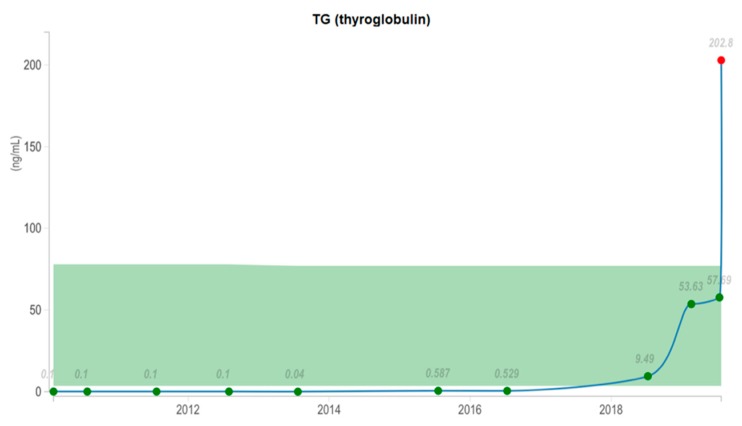
Thyroglobulin (TG) level dynamics (2010–2019). At the ending of 2018 TG level started to rise, in 2019 reaching a maximum level of 202.8 ng/mL.

**Figure 4:**
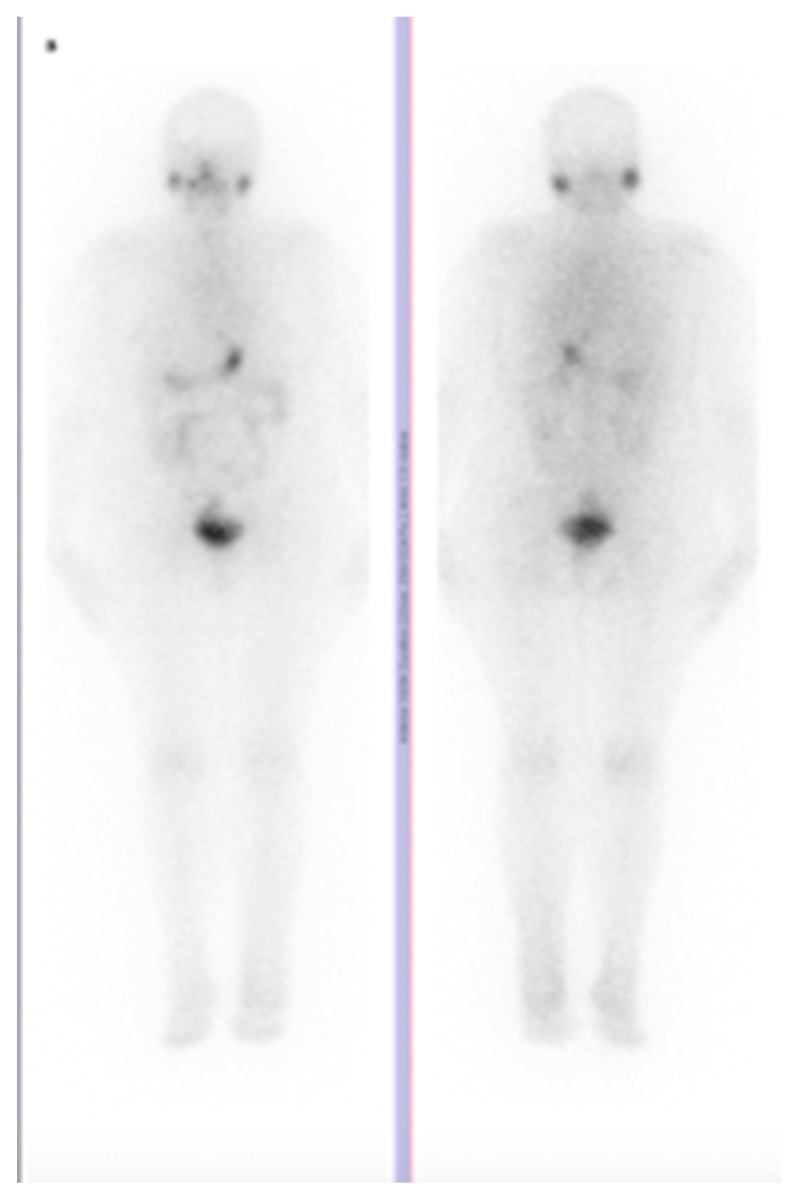
Whole Body Scan with I-131 sodium iodide (I-131 WBS) with 91,4 mCi (3381 GBq) at 48 h postadministration, with no pathological uptake.(anterior, **left**, posterior, **right**) (2019).

**Figure 5:**
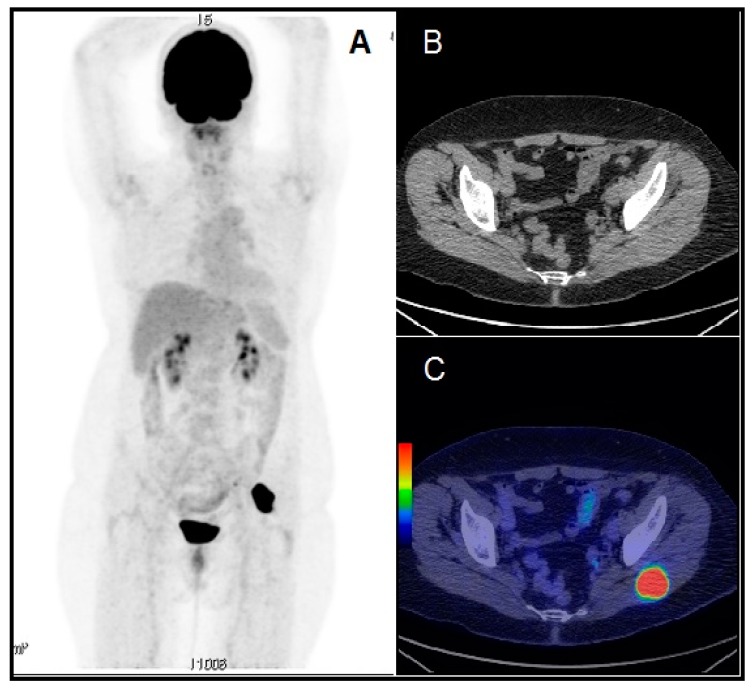
Whole body 18F-FDG PET/CT imaging revealed a right gluteal muscle tumor of 39 × 35 × 41 mm, with intense FDG uptake (SUV lbm max = 16.77) (**A**) 3D-MIP image. (**B**) CT axial image of the gluteal region. (**C**) PET/CT fusion image showing the intense uptake in the left gluteal muscle.

After surgery, her Tg level continued to rise, as in Figure 3, and the patient received a second 3.38 GBq dose of radioactive iodine, with negative post therapy I-131 whole-body scan, as in Figure 4. For further evaluation, we performed a F-18 fluorodeoxyglucose (FDG) positron emission tomography/computer tomography (PET/CT) scan, which showed a 39/35/41 mm tumor in the left gluteal muscle with focal pathological uptake SUV lbm max = 16.77, highly suggestive for a metastatic lesion, as in Figure 5. After surgery and histology exam, the results confirmed papillary thyroid carcinoma metastases.

After surgery, the patient received another I-131 dose of 5.5 GBq, with negative WBS and was submitted to external beam therapy; at the moment of this paper the patient is alive, clinically negative and during radiotherapy.

The other cases presented from the literature had a similar therapeutic approach: radical surgery whenever was possible, radioiodine, in one case being added to systemic therapy with thyrosine kinase inhibitors, in the study of Mohapatra et al.

As a special mention, despite the late recurrence of the disease, presence of distant metastasis and the aggressiveness, the patient clinical status was not significantly altered, and the negative outcome was not an accelerated one.

Usually, papillary microcarcinoma has an excellent prognosis and the current guidelines recommend a less aggressive therapy in the majority of cases. Beyond this commune behavior, there are some cases where the natural history is very aggressive, fact that requires a special attention and a careful evaluation of each case. To our knowledge, there has been only one report in the specialty literature of muscular metastasis from papillary microcarcinoma. Our study underlines the need to evaluate individually and carefully every patient with papillary thyroid microcarcinoma, in order to accurately plan an appropriate therapeutic strategy. The epidemiological aspects of thyroid metastases in rare sites are largely unknown and their identification could have a significant impact on patient management.

## 3. Conclusions

Although papillary thyroid microcarcinoma is the mildest form of all types of thyroid cancer, however, there is a possibility that the behavior of this type of cancer may become aggressive. This is yet another reason why each patient should be treated individually and followed closely. Reporting the course of these rare cases is essential for a better understanding of the pathology and management of future cases.

## Figures and Tables

**Table 1:** Muscle metastases from PTC reported previously in literature.

Author/Origin	Year	Patient Sex/Age	Muscle Involved	Muscle Lesion (nr.)	Other Metastasis
Bae S Y, et al [10]. *Seoul, Korea*	2011	*f*/31	vastus medialis (distal femur)	1	0
Bruglia M, et al [11]. *Ancona, Italy*	2009	*m*/44	biceps femuris	1	lung, mediastinum, brain
Caobelli F, et al [12]. *Brescia, Italy*	2011	*f*/68	right adductor longus; right iliopsoas	2	0
Kuscic L J, et al [13]. *Split, Croatia*	2016	*m*/68	left thigh (medial muscle group)	1	kidney, lung
Li Z G, et al [14]. *Tianjin, China*	2016	*m*/84	bilateral piriformis, left erector spinae, gluteus max.	4	spleen, bones,
Luo Q, et al [15]. *Shanghai, China*	2008	*m*/29	erector spinae	1	kidney, lung
Mohapatra T, et al [16]. *Pradesh, India*	2012	*m*/42	left gluteal, right erector spinae	2	liver
Panoussopoulos D [17], *Athens, Greece*	2007	*f*/69	trapezoid	1	0
Pucci A, et al [18]. *Torino, Italy*	2006	*m*/77	right biceps	1	0
Qiu ZL, et al [19]. *Shanghai, China*	2009	*m*/82	erector spinae	1	manubrium sterni
Sarma M, et al [7]. *Cochin, India*	2014	*m*/66	left deltoid	1	0
Yang J, et al [9]. *Hangzhou, China*	2014	*m*/31	left gastrocnemius	1	lung
Zhao L, et al [20]. *Chengdu, China*	2010	*f*/53	left rectus abdominis	1	0
